# The Old Fashioned Girl

**Published:** 1892-04

**Authors:** 


### THE OLD FASHIONED GIRL.

There is something that is getting to be awfully scarce in this world. Shall I tell you what it is? It is girls. That is what is missing out of the sentient, breathing, living world just now. We have lots of young ladies and lots of society misses, but the sweet, old fashioned girls of ever so long ago are vanished with the poke bonnets and the cinnamon cookies. Let me enumerate a few of the kind of girls that are wanted. In the first place we want home girls—girls who are mother's right hand; girls who can cuddle the little ones next best to mamma, and smooth out the tangles in the domestic skein when things get twisted; girls whom father takes comfort in for something better than beauty, and the big brothers are proud of for something that outranks the ability to dance or shine in society. Next, we want girls of sense—girls who have a standard of their own regardless of conventionalities, and are independent enough to live up to it; girls who simply won't wear a trailing dress on the street to gather up microbes and all sorts of defilement; girls who won't wear a high hat to the theatre, or lacerate their feet and endanger their health with high heels and corsets; girls who will wear what is pretty and becoming and snap their fingers at the dictates of fashions when fashion is horrid and silly. And we want good girls—girls who are sweet, right straight out from the heart to the lips; innocent and pure and simple girls with less knowledge of sin and duplicity and evil-doing at 20 than the pert little school girl of 10 has all too often; girls who say their prayers and read their Bibles and love God and keep his commandments. (We want those girls "awful bad!") And we want careful girls and prudent girls, who think enough of the generous father who toils to maintain them in comfort, and of the gentle mother who denies herself much that they may have so many pretty things, to count the cost and draw the line between the essentials and non-essentials; girls who strive to save and not to spend; girls who are unselfish and eager to be a joy and a comfort in the home rather than an expensive and a useless burden. We want girls with hearts—girls who are full of tenderness and sympathy, with tears that flow for other people's ills, and smiles that light outward their own beautiful thoughts. We have lots of clever girls and brilliant girls, and witty girls. Give us a consignment of jolly girls, warm hearted and impulsive girls; kind and entertaining to their own folks, and with little desire to shine in the garish world. With a few such girls scattered around life would freshen up for all of us, as the weather does under the spell of summer showers. Speed the day when this sort of girl fills the world once more, overrunning the space where God puts them as climbing roses do when they break through the trellis to glimmer and glint above the common highway, a blessing and a boon to all who pass them by.

